# One-Stage Brake Light Status Detection Based on YOLOv8

**DOI:** 10.3390/s23177436

**Published:** 2023-08-25

**Authors:** Geesung Oh, Sejoon Lim

**Affiliations:** 1 Graduate School of Automotive Engineering, Kookmin University, 77, Jeongneung-ro, Seongbuk-gu, Seoul 02707, Republic of Korea; gsethan17@kookmin.ac.kr; 2 Department of Automobile and IT Convergence, Kookmin University, 77, Jeongneung-ro, Seongbuk-gu, Seoul 02707, Republic of Korea

**Keywords:** brake light status, one-stage detection, YOLOv8, ADAS, autonomous

## Abstract

Despite the advancement of advanced driver assistance systems (ADAS) and autonomous driving systems, surpassing the threshold of level 3 of driving automation remains a challenging task. Level 3 of driving automation requires assuming full responsibility for the vehicle’s actions, necessitating the acquisition of safer and more interpretable cues. To approach level 3, we propose a novel method for detecting driving vehicles and their brake light status, which is a crucial visual cue relied upon by human drivers. Our proposal consists of two main components. First, we introduce a fast and accurate one-stage brake light status detection network based on YOLOv8. Through transfer learning using a custom dataset, we enable YOLOv8 not only to detect the driving vehicle, but also to determine its brake light status. Furthermore, we present the publicly available custom dataset, which includes over 11,000 forward images along with manual annotations. We evaluate the performance of our proposed method in terms of detection accuracy and inference time on an edge device. The experimental results demonstrate high detection performance with an mAP50 (mean average precision at IoU threshold of 0.50) ranging from 0.766 to 0.793 on the test dataset, along with a short inference time of 133.30 ms on the Jetson Nano device. In conclusion, our proposed method achieves high accuracy and fast inference time in detecting brake light status. This contribution effectively improves safety, interpretability, and comfortability by providing valuable input information for ADAS and autonomous driving technologies.

## 1. Introduction

In the past decade, significant progress has been made in the development of Advanced Driver Assistance Systems (ADAS) and autonomous driving technologies. These technologies have become increasingly intelligent, with autonomous driving progressing beyond level 2 toward level 3 of driving automation, as defined by the Society for Automotive Engineers (SAE) [1]. However, the mass production of technology surpassing level 3 of driving automation is not yet available, primarily due to three main reasons as follows: safety, interpretability, and ride comfort. In level 3, the system must be liable for all potential accidents that occur during its operation, making safety a top priority. To ensure safety, it is crucial to redundantly acquire various forms of perception information. Claiming responsibility necessitates providing justifications for the system’s perception information, while highlighting the importance of ensuring interpretability of the perception information is also essential. In addition to quantitative perception information, such as distance to preceding vehicles, relative velocity, and time to collision (TTC), more intuitive information is needed. Furthermore, ride comport is a significant issue that should not be overlooked in the widespread adoption of ADAS and autonomous driving technology, with motion sickness being a crucial concern [2,3]. Some even refer to it as “autonomous car sickness” [3]. Motion sickness arises from conflicts between sensory inputs, where the detected motion information deviates from the expectations based on past experiences during autonomous driving [4,5]. In other words, autonomous driving systems need to mimic human driving behavior to mitigate motion sickness and enhance ride comfort.

To address these challenges, we propose a new method for detecting driving vehicles and their brake light status. Brake light status information is crucial for multiple drivers to ensure safety on the road. Human drivers adjust their driving patterns based not only on distance and relative velocity but also on the brake light status of neighboring vehicles. Autonomous driving systems aiming for enhanced safety should not overlook this fundamental information. Additionally, brake light status is particularly intuitive and possesses effective interpretability when communicating with humans. While distance, relative velocity, and TTC may provide clearer information among machines, brake light status information offers a more intuitive understanding. For example, when following a leading vehicle, a machine can maintain a much tighter gap with the leading vehicle by utilizing a combination of distance, relative velocity, and TTC than human drivers. However, since human drivers normally cannot grasp the intricacies of distance, relative velocity, and TTC as tightly as the machines do, when the brake light of the leading vehicle illuminate, human drivers stop acceleration and prepare for possible deceleration of the leading vehicle. Therefore, driving technology that considers brake light status as well can be more persuasive to humans. Furthermore, considering the brake light status in ADAS can improve ride comfort [6]. By acquiring redundantly interpretable perceptual information, including brake light status, ADAS (or autonomous driving systems) can drive in a more human-like manner, ultimately reducing sensory conflicts and alleviating motion sickness.

**Figure 1:**
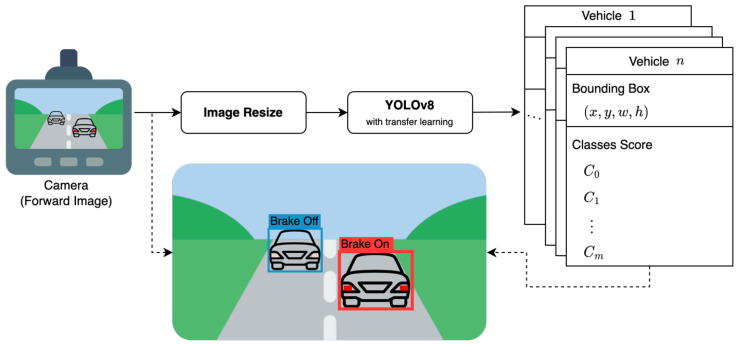
Workflow of the proposed one-stage brake light detection network. The solid line arrows represent the flow of data for inference purposes, while the dashed line arrows represent the flow of data for visualization purposes.

In this paper, we propose a one-stage neural network that detects the brake lights of preceding vehicles, taking into account the trade-off between inference time and accurate detection performance. The proposed network is based on YOLOv8 [7], the latest version of the popular one-stage object detection network, as shown in Figure 1. We leverage transfer learning to train the network for the task of detecting driving vehicles and brake light.

To train the network, we collected over 11,000 real-world driving images and manually annotated the driving vehicle and brake light status for each image. This dataset was used for transfer learning with YOLOv8. We conducted transfer learning and evaluated the performance of the models in terms of model size, brake light status, and ambient lighting conditions. The real-time performance of the trained models was verified using an edge device, the Nvidia Jetson Nano, installed in the driving vehicle. The Jetson Nano is one of Nvidia’s platform series designed for embedded applications of neural networks. Among the product line, it stands out as the smallest form factor with the lowest power consumption, making it widely used for real-time inference experiments, especially in resource-constrained environments [8]. This verification allowed us to assess the model’s ability to make predictions in real-time while considering computational efficiency.

The main contributions in this study can be described as follows:

- Proposal of a one-stage network for detecting the brake light status of vehicles in the forward image during driving. The proposed network takes a single image as input and provides bounding boxes for all vehicles in the image, along with the detection of whether their brake light is on or off.
- Introduction of a dataset specifically designed for the task of driving vehicle and brake light status detection. The dataset comprises over 11,000 real-world driving images captured under various conditions, including day, night, and tunnel scenarios. Each image in the dataset is annotated by experts with vehicle-bounding boxes and brake light status.
- Fine-tuning of the proposed detection network based on YOLOv8 using the introduced dataset. The trained model demonstrates high detection performance, and its real-time performance on an edge device is validated.

The remainder of this paper is organized as follows: Section 2 provides an overview of related works on brake light status detection. Section 3 discusses the proposed detection network and dataset used for transfer learning. Section 4 presents the methodology for transfer learning and analyzes the evaluation results. Section 5 concludes the work and outlines potential future research directions.

## 2. Related Works

Research has been conducted on vehicle brake light status detection for various purposes, including collision avoidance and deceleration prediction. These studies primarily rely on forward images, as the brake light’s brightness difference serves as the most significant clue. Detection methods can be broadly categorized into image processing [9,10,11], frequency-tuned [12], machine learning [13,14,15], and deep learning approaches [16,17,18]. In terms of research scope, most studies focus on daytime conditions [10,11,13,14,15,16], targeting scenarios where the detection of brake light status is relatively straightforward. However, there are also studies that address more challenging scenarios, such as nighttime [9,12] or tunnel environments [18], where the detection becomes more difficult due to potential confusion with tail lights. It is less common to find studies that simultaneously address both day- and nighttime conditions [17].

Studies that utilize image processing techniques employ heuristic approaches and leverage different color spaces for brake light detection. Liu et al. utilized the most common red-green-blue (RGB) color space and apply a threshold for color difference between adjacent frames to detect brake light operation [11]. Similarly, Thammakaroon and Tangamchit also used a threshold in the RGB space, but they additionally performed low-light image processing in the hue-saturation-intensity (HSI) color space, focusing on detecting brake light operation in nighttime images [9]. Chen et al. utilized the a* component in the LAB color space to perform binary thresholding for brake light detection [10]. In the LAB color space, L* represents the lightness, while a* and b* represent the color ranges, with a* representing the red–green axis and b* representing the yellow–blue axis. These different color spaces are also utilized in the image preprocessing stages of studies that employ machine learning or deep learning approaches. Cui et al. used the hue-saturation-value (HSV) color space [13], while Nava et al. and Pirhonen et al. utilized the LAB color space [14,15].

In contrast to the heuristic approaches mentioned earlier, Chen and Peng focused on finding invariant features in the frequency domain, presenting an effective methodology for extensive datasets [12]. However, in recent years, learning-based methods have shown much more effective performance, leading to extensive research on brake light detection based on machine learning or deep learning techniques. Cui et al. and Nava et al. achieved high brake light detection performance using support vector machines (SVM), with a 75% detection rate and an F1 score of 0.95, respectively [13,14]. Pirhonen et al. achieved an accuracy of 82% using the random forest, primarily focusing on objects at distances of 50 m or more [15]. Wang et al. utilized deep learning methods, specifically convolutional neural networks (CNN), as the foundation for detecting brake light status and other related features [16]. They used a pre-trained AlexNet model [19] from the ImageNet dataset [20], and achieved an accuracy of 89%. Kim utilized not only CNN but also long short-term memory (LSTM) to detect the brake light status of driving vehicles, achieving an accuracy of 90.2% specifically for vehicles driving inside tunnels [18]. While these machine learning and deep learning methods demonstrated high performance, it is important to note that they all have limitations in that they primarily consider specific scenarios, such as daytime or tunnels. Additionally, they have been validated only on their own nonpublic datasets.

A common limitation among all the mentioned brake light detection studies is the use of a multi-stage detection structure. These methods all assume that the target vehicle has been detected beforehand, and then the detection of the brake light is performed. Various methods are used for target vehicle detection, such as the histogram of oriented gradients (HOG) detector [10,16], combination of HOG detector with SVM [13], and AdaBoost [11,21]. Additionally, initial versions of YOLO [22,23] and improved versions such as YOLOv3 [24] and YOLOv4 [25] are employed for driving vehicle detection [15,18]. Accurate detection of driving vehicles can enhance the performance of brake light status detection. However, the multi-stage approach has a significant limitation in terms of real-time capability. The fundamental purpose of brake light status detection is to contribute to the development of safer systems by integrating with ADAS or autonomous driving systems. If real-time performance is not ensured, even highly accurate detection loses its value.

Li et al. proposed a one-stage detector for real-time brake light status detection [17]. They utilized a light version of YOLOv3, called YOLOv3-tiny, as the backbone network, and enhanced the detection performance by adding output layers and spatial pyramid pooling (SPP [26]). They reported a detection performance of mAP 89.13 for brake activation detection and achieved real-time capability with a frame rate of 63 FPS. However, it is important to note that the reported frame rate was measured on a powerful GPU (GTX-1060), which is significantly more powerful than the hardware typically used in real-world vehicle control systems. Therefore, it is necessary to propose brake light status detection models that can achieve high detection performance with fast inference speed on the edge devices, which are commonly used in real-world vehicle control systems.

## 3. Proposed Work

In this study, we propose a one-stage brake light detection network based on YOLOv8. The network is designed to detect both the driving vehicle and its brake light status in a single stage. The input to the network is a single forward image captured from the ego-vehicle, and the outputs consist of the brake light status categories and 2D bounding boxes for all driving vehicles present in the input image (as depicted in in Figure 1). The 2D bounding box is represented by four numerical values: *x*, *y*, *w*, and *h*. The coordinates (x,y) represent the center of the bounding box, while *w* and *h* denote the width and height of the bounding box, respectively. The number of classes, denoted as *m*, is defined as 2, including the brake light status categories. The classes score, denoted as *C*, represent the probability value associated with each category:

(1) $$m=2,\;\;\;C\left\{\begin{array}{cc} C_{1}=1.0 & \;\mathrm{If}\;\mathrm{the}\;\mathrm{brake}\;\mathrm{light}\;\mathrm{is}\;\mathrm{turned}\;\mathrm{off}\;\\ C_{2}=1.0 & \;\mathrm{If}\;\mathrm{the}\;\mathrm{brake}\;\mathrm{light}\;\mathrm{is}\;\mathrm{turned}\;\mathrm{on}\; \end{array}\right.,\;w.r.t\sum_{i=1}^{m}C_{i}=1.0$$

where $C_{1}$ is the probability that the brake light is turned off and $C_{2}$ is the probability that the brake light is turned on of the detected vehicle. Hence, the proposed network outputs the bounding boxes for the target vehicles present in the input image, along with the corresponding probability values for the two defined classes. The target vehicles include conventional passenger cars as well as motorcycles, buses, trucks, and special vehicles. Only the vehicles with two or more functional brake lights on both the left and right sides of their rear end are taken into consideration as target vehicles.

### 3.1. Dataset

To accomplish the specific task of detecting brake light status along with the vehicle, a custom dataset needs to be prepared. The process of creating a custom dataset involves collecting input images and annotating them accordingly. To collect forward images of the driving vehicle, we utilize a dashboard camera specifically designed to capture video footage of the road ahead during driving. We employ two different cameras, namely the FINEVu GX2000 and Mercedes-Benz dashcam, which are mounted on the windshields of different vehicles. After driving with the vehicle equipped with the dashboard camera, we extract single images from the recorded video. However, considering that the dashboard camera typically captures more than 30 images per second, using every single image as input for the dataset would result in a large number of similar images. To address this issue and prevent redundancy in the dataset, we collect the input images at *T*-second intervals from the video.

Another crucial aspect to consider when utilizing dashboard camera images is the camera’s image postprocessing capabilities. Dashboard camera often include features such as brightness correction and High Dynamic Range (HDR) to capture clear details, especially in critical situations such as car accidents. However, it is essential to account for the preprocessing of input data in training a network that can deliver reliable performance even with a general camera lacking image postprocessing features. The data preprocessing methods we consider are described in Section 4.1.

**Figure 2:**
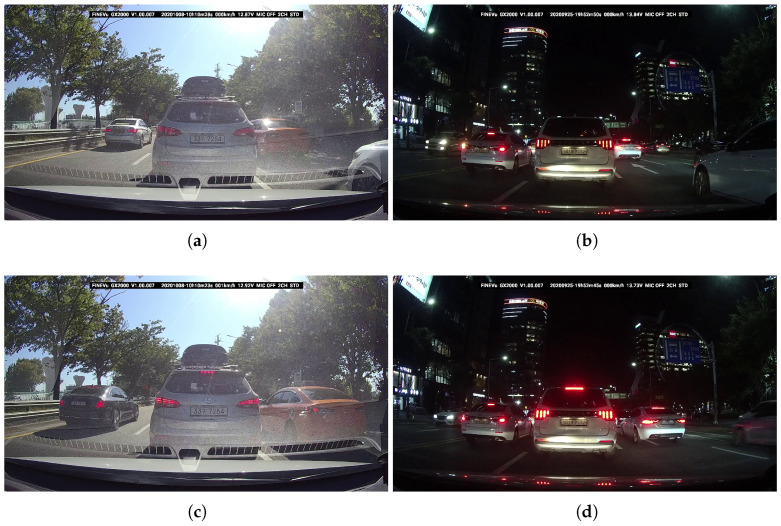
Illustrative cases in which it is difficult to determine the operation of brake lights based on single image. (**a**) Light reflection from LED lights. (**b**) Confusion with tail lights. (**c**) Preceding image of (**a**). (**d**) Preceding image of (**b**).

For supervised transfer learning, annotation information in the same format as the output of the proposed network is required. Six annotation experts manually label information such as the bounding box (x,y,w,h) and brake light status (*C*) of vehicles appearing in each collected image. The experts employ the widely used open-source image annotation tool called LabelImg. This tool has become part of the open-source data labeling tool, Label Studio [27], which provides more flexible functionalities. There are several factors that make it challenging for trained experts to annotate from a single image, even with the assistance of useful tools. One prominent challenge is the presence of light reflection from Light Emitting Diode (LED) lights and confusion with tail lights. Since most vehicle brake lights are composed of LED lights, they can easily reflect ambient light. Consequently, when ambient light is reflected from an LED light and reaches the camera, the LED itself may appear to emit light even if it is not turned on. This illusion creates difficulty in determining whether the brake light of a vehicle is turned on or off. Figure 2a illustrates a scenario where it is challenging to discern the brake light status of the vehicle in the center of the image due to the bright surrounding light. Even when comparing the light intensity of the vehicle on the left side of the image, where the brake light is turned on, with the vehicle on the right side of the image, where the brake light is turned off, it shows an intermediate level. While the light reflection is more prevalent during the daytime, when there is ample ambient light, confusion with tail lights arises at night. Figure 2b showcases a scenario where it is difficult to determine whether the vehicle in the center of the image has only the tail light of both the tail light and brake light is turned on. This confusion becomes more pronounced when there are no surrounding vehicles in the image. To overcome these challenges, the experts annotate by referring to several preceding or succeeding images. Figure 2c,d depict preceding images captured in close proximity to Figure 2a,b, respectively. By referring to the preceding images, it becomes much easier to determine whether the brake lights of the vehicles in Figure 2a,b are turned on.

The details of the dataset are described in Section 4.1, and the train dataset is publicly available for access [28].

### 3.2. YOLOv8

YOLO, which stands for “You Only Look Once”, is a well-known multi-object detection algorithm [22]. As its name suggests, YOLO aims to provide detection results by analyzing the image only once. Prior to the introduction of YOLO, many multi-object detection algorithms relied on multiple stages to accurately detect object location and class [26,29,30,31]. However, these approaches had limitations in real-time applications due to the need for multiple steps. YOLO revolutionized object detection by simultaneously detecting the locations and classes of objects using a single neural network. Since its inception, YOLO has been recognized for its fast inference speed and high accuracy compared to other object detection algorithms. It has evolved from YOLOv1 to the latest state-of-the-art version, YOLOv8 [7,22,23,24,25,32,33,34]. YOLO has extended its capabilities beyond its original task of detecting 80 objects defined by MS-COCO [35] and has been widely utilized as a backbone or benchmarking model in various detection fields, including remote sensing [36] and tiny defects detection [37]. It has provided significant research inspiration across different domains. Therefore, in this study, we propose a network based on YOLOv8, the latest state-of-the-art one-stage multi-object detection algorithm, to detect driving vehicle on the road and their brake light status, building upon the rich foundation of YOLO’s achievements in diverse detection tasks.

YOLOv8 [7] was officially released in January 2023 and incorporates updates from YOLOv5 [32]. Notable updates in YOLOv8 include structural changes in the partial bottleneck, a shift to an anchor-free approach with the decoupled head, and a change in the activation function of the top layer [38]. The loss functions utilized in YOLOv8 include binary cross-entropy for classification loss, complete intersection over union (CIoU) [39], and distribution focal loss (DFL) [40] for localization loss. The output of the proposed network consists of 8400 bounding boxes, with 6 parameters assigned to each input image. These parameters represent the 2D center coordinates, width, and height of the bounding box (x,y,w,h), as well as the probability values for each class, indicating whether the brake light is turned off or on. Among the 8400 output bounding boxes, postprocessing techniques such as nonmaximum suppression (NMS) are employed to filter out insignificant detections. This helps eliminate redundant and overlapping bounding boxes, resulting in a more refined and accurate set of detections.

## 4. Experiments

The experimental evaluation of the proposed method is presented in this section. Section 4.1 provides details about the custom dataset collected as described in Section 3, as well as preprocessing steps. Section 4.2 focuses on the transfer learning process of YOLOv8 using the custom dataset. The experimental results are presented in detail in Section 4.3.

### 4.1. Custom Dataset and Preprocessing

A large-scale custom dataset consisting of over 16 h of data was collected. Videos recorded from dashboard cameras during real road driving were utilized, resulting in a total of more than 11,000 images. These images were obtained by setting the time interval *T* to 5 s. Manual annotation was performed on all images to label the bounding box of the driving vehicle and the brake light status of each vehicle. The total number of annotations exceeded 30,000. To ensure a balanced and diverse dataset, various driving scenarios were included, such as daytime, nighttime, city, highway, and tunnel environments. Precautions were taken to avoid bias toward any specific category, ensuring information about the number of images and annotations in the train and test sets for each category of brake light status. The details of numbers of images and annotations in the train and test sets in each category of brake light status are given in Table 1.

To make our custom dataset trainable with YOLOv8 and achieve robust performance, several preprocessing steps are necessary. The first crucial processes involve image resizing and normalization. In order to maintain a consistent input size, the width and height of all images were resized to $I_{w}$ and $I_{h}$, respectively. In this study, both $I_{w}$ and $I_{h}$ were defined as 640. Regarding image normalization, min-max normalization was applied to normalize the pixel values. The normalization process ensures that all pixel values fall within a specific range, typically between 0 and 1, and is performed as follows:

(2) $$x_{norm}=\frac{x-x_{min}}{x_{max}-x_{min}}$$

where *x* and $x_{norm}$ are the origin and normalized pixel value, respectively, and $x_{max}$ and $x_{min}$ are the maximum and minimum pixel value of the image, respectively. In this study, the values for $x_{max}$ and $x_{min}$ were set to 255.0 and 0.0, respectively, following the usual convention. Furthermore, various data augmentation techniques were applied to enhance the robustness of the inference performance. Random horizontal flipping and image cropping were performed to generate variations of the collected images that could realistically occur. To improve detection performance in cases of occlusion, random black-box cutout augmentation was also applied. Finally, to ensure robustness across different camera setups, the quality of the input images was intentionally degraded using various methods. As stated in Section 3.1, the dashboard cameras used for image acquisition are equipped with various postprocessing methods to captured high-quality images. To ensure the robust performance of the trained network even with general cameras, random modifications, such as brightness changes, blur, and noise injection, were applied to the images. The details of all random modifications applied during the preprocessing stage are as follows:

- Crop: zoom rate chosen from uniform distribution within the range of 0% to 20%;
- Cutout: a maximum size of black-box is 20% of image size, a maximum of 3 boxes;
- Brightness: adjustment with a range of minimum −25% to maximum +25%;
- Blur: a gaussian blur with a maximum kernel size of 3×3;
- Noise: add noise to a maximum of 5% of pixels.

**Figure 3:**
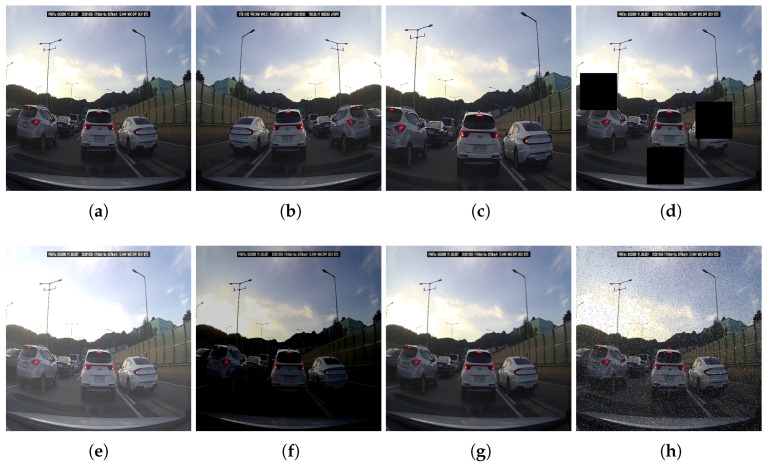
Examples of individual random modifications applied during the preprocessing stage. (**c**–**h**) represent example images that reflect the maximum extent of each random modification considered. (**a**) Reshape. (**b**) Horizontal flip. (**c**) Crop. (**d**) Black-box cutout. (**e**) Brightness increase. (**f**) Brightness decrease. (**g**) Blur. (**h**) Noise.

**Figure 4:**
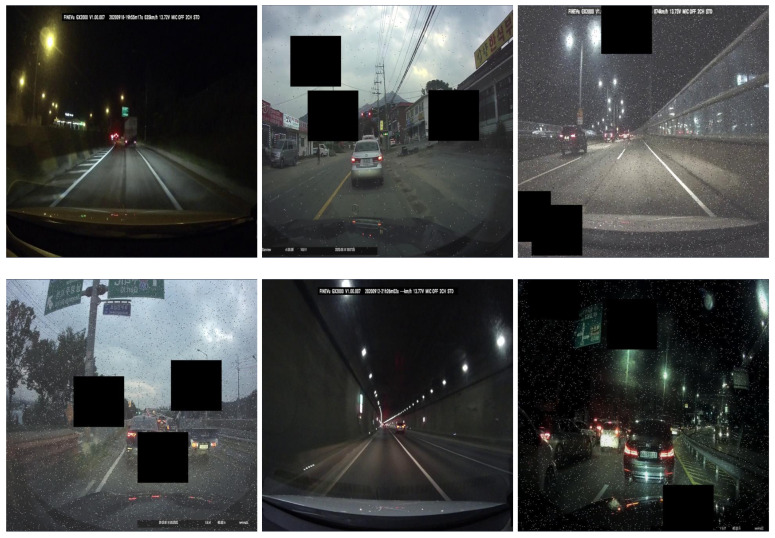
Preprocessed input images of the custom dataset.

The images illustrating each modification can be found in Figure 3. Figure 4 provides examples of preprocessed images. All preprocessing steps were performed using Roboflow [41], a comprehensive platform for computer vision and image processing tasks. The preprocessed train dataset is publicly available for access [28].

### 4.2. Transfer Learning

In this study, we conducted transfer learning on all models provided by YOLOv8 [7] to develop a detection network that achieves real-time inference in a driving vehicle while maintaining accurate detection. The YOLOv8 architecture offers five different models of varying sizes, ranging from the smallest model (YOLOv8n) to the largest model (YOLOv8x), as shown in Table 2. To ensure consistency, the same set of hyperparameters was applied to all models during training. A random 20% of the train dataset was set aside as the validation dataset for monitoring the training progress. The initial parameter values for each model were obtained from the pretrained parameters officially provided by YOLOv8. The training process consisted fo 300 iterations, with a patience value of 20. If there was no observable improvement in the validation loss over the most recent 20 iterations, the training was terminated early to save time and resources. Stochastic gradient descent (SGD) optimizer with a learning rate of 0.01 was used, incorporating both momentum and Nesterov Accelerated Gradient (NAG) techniques [42] with a momentum coefficient of 0.937. The training loss was calculated using three loss function: binary cross-entropy, CIoU, and DFL. The weighting factors assigned these loss functions were 0.5, 7.5, and 1.5, respectively. These factors were chosen to balance the impact of each loss function during training.

### 4.3. Results

**Figure 5:**
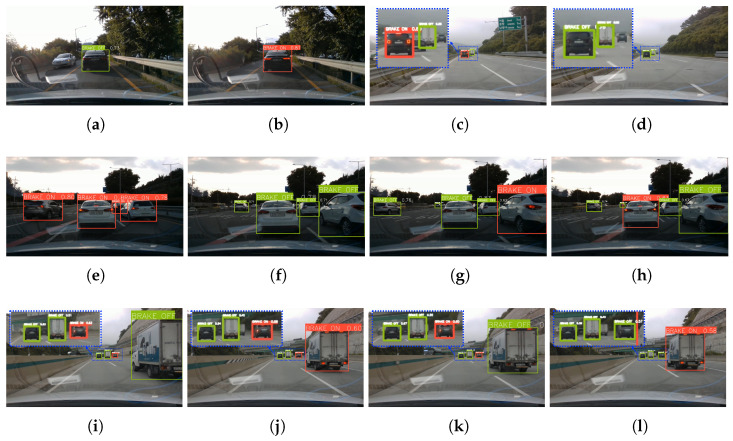
Qualitative analysis images of driving vehicle and brake light status detection by proposed networks. (**a**,**b**) is a continuous image pair showcasing the scenario with leading vehicle at a close distance. (**c**,**d**) is the continuous image pair showcasing the scenario with leading vehicle at a far distance. (**e**–**h**) are the continuous image pairs showcasing the scenario with multiple vehicles at a close distance. (**i**–**l**) are the continuous image pairs showcasing the scenario with multiple vehicles at a far distance.

In this section, the evaluation results of the trained detection models are presented, both qualitatively and quantitatively. Qualitative analysis confirmed that the trained detection models accurately detect the bounding boxes of driving vehicles and classify their brake light status (on or off) in various road environments. Figure 5 shows some of the images used for qualitative analysis. These images are provided as pairs of two or more consecutive frames to demonstrate clear analysis results. Figure 5a,b represent one continuous image pair displaying the detection performance of leading vehicle located at a close distance. Figure 5c,d represent another continuous image pair displaying the detection performance of leading vehicle located at a far distance. As evident from these two image pairs, the proposed model accurately detects the location and brake light status of leading vehicles regardless of their distance from the ego vehicle. Figure 5e–h represent other continuous image pairs depicting scenarios with multiple vehicles present at a close distance. In these images, the location and brake light status of all vehicles in the images are successfully detected. Furthermore, even in scenarios with multiple vehicles at a far distance, all vehicles are accurately detected, as demonstrated by images pairs Figure 5i–l.

**Figure 6:**
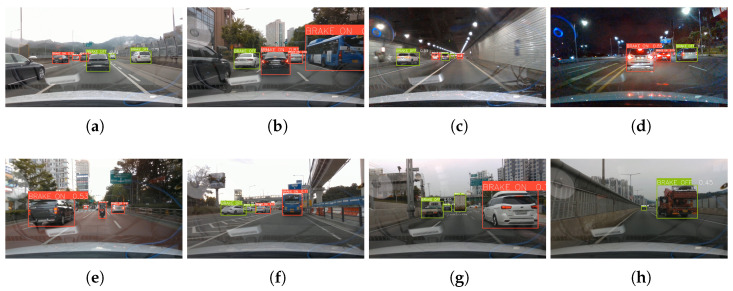
Qualitative analysis images for various driving environments and vehicle types, mainly intended as examples for the following: (**a**) Hightway, (**b**) City, (**c**) Tunnel, (**d**) Nighttime, (**e**) Motorcycle, (**f**) Bus, (**g**) Truck, and (**h**) Special vehicle.

Qualitative analysis was conducted not only for different vehicle quantities and distances but also for various driving environments and vehicle types. Figure 6a–d demonstrate the robust performance of the trained model in diverse driving environments, including highway, city, tunnel, and nighttime. Figure 6e–h provide qualitative evidence that the model has ability to detect various vehicle types, including passenger cars, motorcycles, buses, trucks, and special vehicles.

The evaluation of the driving vehicle and brake light status detection performance of each model that underwent transfer learning is conducted by calculating the mean average precision (mAP) on the test set. Two mAP values are calculated: mAP50 and mAP50-95. mAP50 represents the average precision at an intersection over union (IoU) threshold of 0.5. The IoU threshold measures the overlap between the predicted bounding boxes and the ground truth labels, indicating how well the predicted boxes align with the actual objects. mAP50-95 represents the average precision over a range of IoU thresholds from 0.5 to 0.95, with a step size of 0.05. This metric provides comprehensive assessment of the detection model’s performance across different levels of overlap. Both mAP50 and mAP50-95 are commonly used metrics to evaluate the overall performance of object detection models.

**Figure 7:**
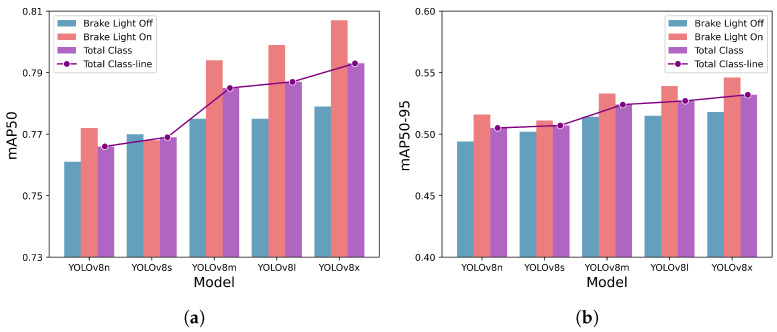
Detection performance on the entire test set. The *y*-axis represents the detection accuracy, and the *x*-axis lists the YOLOv8 models, with larger models positioned toward the right. Each model is depicted with three bars, showcasing the detection performance for brake light off class, brake light on class, and total class, respectively. The single line plot highlights the difference in detection performance by models for total class. (**a**) mAP50 on the entire test set. (**b**) mAP50-95 on the entire test set.

Figure 7 presents the detection performance of each trained model, showcasing the results for mAP50 and mAP50-90 in Figure 7a,b, respectively. The detection performance for each individual classes, brake light off and brake light on, is represented by blue and red bars, respectively. The overall detection performance for all classes is shown by the purple bar, with a purple line plot illustrating the trend of performance differences across models. Both mAP50 and mAP50-95 exhibit similar overall trends, although they differ in scale. As expected, the detection performance for all classes generally increases as the model size increases. However, the YOLOv8s model shows a slightly lower performance increase, primarily due to its lower brake light on class detection performance. Comparing the mAP50 values for each class, it can be observed that all models, except for YOLOv8s, have higher detection performance for the brake light on class compared to the brake light off class. Overall, the proposed methodology achieved mAP50 values ranging from 0.766 to 0.793 and mAP50-95 values ranging from 0.505 to 0.532. Considering the recent benchmarking performance of MS-COCO [35], which is one of the leading object detection, with mAP50 values ranging form 71.9 to 77.0 and mAP50-95 values ranging from 57.7 to 58.8, the proposed methodology demonstrates significant results [43,44]. Detailed detection performance for each model and class can be found in Table 3.

In Figure 7, it was observed that the brake light on detection performance for the brake light on class was generally better than that for the brake light off. However, since the two classes are distinguished solely based on the brightness of the brake light and not the shape or form of the vehicle, it is possible to hypothesize that ambient illumination can affect the detection performance. To verify this hypothesis, the test dataset was split into two types based on ambient light levels: Day, representing images taken during daytime with high ambient illumination, and Night, representing images taken at night or in tunnels with low ambient illumination. The number of images and annotations for each type are provided in Table 4.

**Figure 8:**
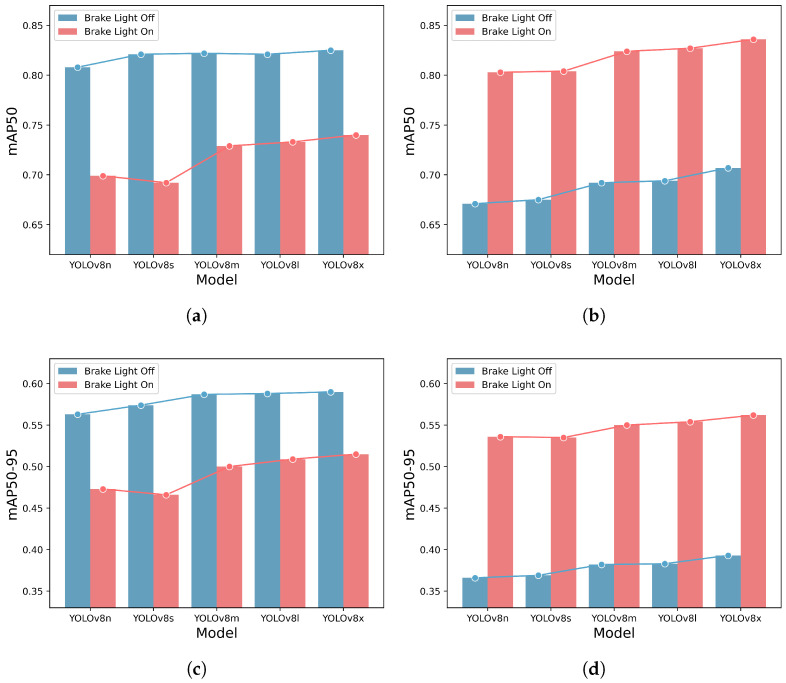
Detection performance comparison for ambient illumination difference. The *y*-axis and *x*-axis represent the detection accuracy and lists the YOLOv8 models, respectively. Each model is depicted with two bars, showcasing the detection performance for brake light off and brake light on classes. Each line plots highlights the difference in detection performance by models for each class. (**a**) mAP50 on the Day test set. (**b**) mAP50 on the Night test set. (**c**) mAP50-95 on the Day test set. (**d**) mAP50-95 on the Night test set.

Figure 8 depicts the detection performance for each class on the Day/Night split test dataset. In Figure 8a,b, mAP50 is plotted, while in Figure 8c,d, mAP50-95 is plotted. Figure 8a,c show the performance on the Day test set, while b,d show the performance on the Night test set. On the Day test set, the detection performance for the brake light off class is better than that for the brake light on class. Conversely, on the Night test set, the detection performance for the brake light on class is superior to that for the brake light off class. The brake light off class, which was well detected in an environment with high ambient light, experienced a decline in detection performance as the ambient light decreased. On the other hand, the brake light on class, which initially exhibited relatively low detection performance in an environment with high ambient light, demonstrated high detection performance when the ambient light was low. The difference in performance due to ambient illumination is more pronounced in the brake light off class. Detailed detection performance comparisons for ambient illumination difference for each model and class can be found in Table 5.

According to the detailed analysis, the performance difference attributed to the difference in ambient illumination can be explained in terms of accuracy. The overall accuracies for driving vehicle detection across all classes were 0.87 in the Day test set and 0.89 in the Night test set. As the ambient illumination decreased, the accuracy for driving vehicle detection slightly improved. However, the accuracies for the brake light off class decreased to 0.67 in the Day test set and 0.43 in the Night test set, while the accuracies for the brake light on class increased to 0.75 in the Day test set and 0.86 in the Night test set. The detailed analysis revealed that this performance difference is influenced by the presence of tail lights. As the ambient illumination decreases, the tail lights of the vehicles are turned on, enhancing the detection performance for driving vehicles. However, the turned-on tail lights can cause confusion with the turned-on brake lights, leading to a decrease in the detection performance for brake light off class. Consequently, the decrease in ambient illumination improves the detection performance of vehicles with the brake light turned on while deteriorating the detection performance of vehicles with the brake light turned off.

**Figure 9:**
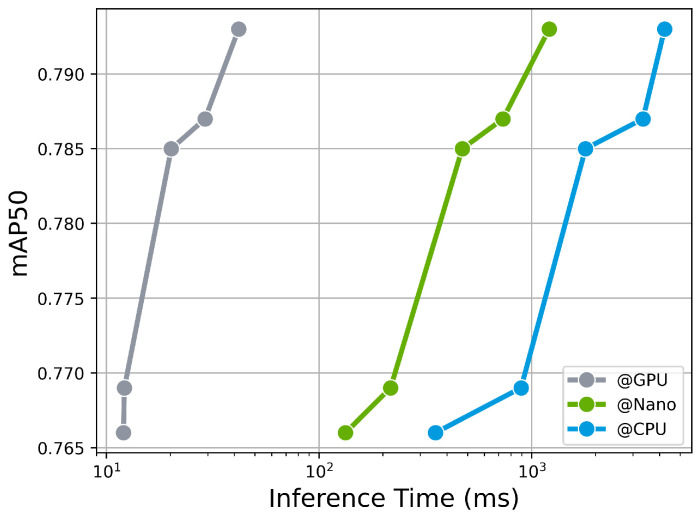
Trade-off between inference time and detection accuracy in different environments. The *x*-axis represents the inference time and the *y*-axis represents the detection accuracy as mAP50. It showcases the trends in inference speed and detection performance for different sizes of YOLOv8 under different computing environments as follows: “@GPU” refers to the Nvidia Tesla T4, “@Nano” refers to the Nvidia Jetson Nano, and “@CPU” refers to the Intel Xeon processor.

In order to validate the real-time inference performance of the trained models on edge devices, experiments were conducted to evaluated both accurate detection and inference time. The Nvidia Jetson Nano device was utilized for this purpose. The trained models were converted to the Open Neural Network Exchange (ONNX) format, which is an open format that facilitates the sharing and interoperability of neural network models across different frameworks. The inference time was measured on the Jetson Nano device using the ONNX models. The measured inference times ranged from 133.30 ms to 733.27 ms, depending on the size of the model. As expected, among the proposed models, YOLOv8n, with the smallest number of parameters and computations, exhibited the fastest inference time of 133.30 ms, surpassing even human cognitive processing time. It is worth noting that the average human cognitive response time is approximately 200 ms. While faster inference time are generally preferred, it is crucial to acknowledge that Jetson Nano operates in a resource-constrained environment. Despite these strict limitations, YOLOv8n achieved inference time faster than human cognitive processing, indicating that it has sufficient real-time capability. The trade-off performance between inference time and detection accuracy is illustrated in Figure 9. To provide a comprehensive performance comparison, the performance on different devices, including the Jetson Nano, CPU (Intel Xeon 2.20 GHz), and GPU (Nvidia Tesla T4), was included. Detailed values can be found in Table 6.

Table 7 provides a detailed description of the differences between our proposed model and the key existing brake light status detection studies. The algorithms listed in Table 7 are state-of-the-art learning-based brake light status detection algorithms. The five studies listed at the top are divided into two or more stages, involving vehicle localization and classification of brake light status [13,14,15,16,18]. The methodologies for each stage are sequentially presented under the second column, named proposed work. It is important to note that these five studies only present evaluation results for brake light status classification, excluding vehicle localization, and hence, evaluation metrics such as detection rate, F1 score, and accuracy were used to describe the classification performance. On the other hand, both the algorithms presented by Li et al. [17] and our proposed algorithm perform vehicle localization and brake light status classification simultaneously in a single-stage process. The evaluation results provided encompass both vehicle localization and brake light status classification, and mAP50 was used to describe both classification and localization performance.

When comparing the two mAP50 values in Table 7, Li et al.’s is higher than ours. However, one should be mindful that the number of classification classes and the dataset differ. The 0.89 value reported by Li et al. [17] pertains only to the performance considering the turned on brake light status, while our value of 0.76 accounts for both turned on and off brake light status. In terms of data and sample size comparison, our research utilized the largest dataset. Furthermore, our dataset includes diverse environmental conditions, including daytime, nighttime, and tunnel scenarios, which were not all simultaneously considered in the other studies. By integrating this vast amount data of diverse conditions, our experimental results effectively represent a wide range of real-world scenarios.

Among the existing key algorithms in Table 7, the inference time of the algorithms is only presented through the study by Li et al. [17], which reported an impressive inference time of 15.87 ms. However, it is important to note that their experiments were conducted on a high-performance GPU, GTX-1060, which may have contributed to the rapid inference speed. In contrast, our study not only utilized a powerful GPU, but also conducted experiments on an edge device with limited resources. As the experimental environments differed, a direct comparison of the inference speeds between the two algorithms is not feasible. Nonetheless, our study presented the inference time on edge device, showcasing real-time performance. This demonstration yields more practical research results, considering the constraints of edge devices and emphasizing the relevance of our findings for real-world applications.

## 5. Conclusions

In this paper, we have proposed an algorithm that utilizes transfer learning with YOLOv8 for the detecting of driving vehicles on the road and their brake light status. Our proposed approach offers novelty in three main aspects. First, we have constructed and publicly released a dataset specifically designed for detecting driving vehicles and brake light status on the road. Acquiring high-quality datasets remains a challenging task, and by making our dataset and annotation results accessible to everyone, we have provided a foundation for related research. Second, we have proposed a one-stage brake light detection network that ensures both high accuracy and fast inference speed. This network, trained with a dataset using YOLOv8, takes a single forward image of a driving vehicle as input and detects all driving vehicles in the image while classifying their brake light status as off or on. Through training and evaluation of models of various sizes, we have achieved high accuracy with a maximum mAP50 of 0.793. We have also provided detailed analysis considering various driving environments, including different ambient illumination conditions. Lastly, we have validated the real-time capability of the proposed network by examining the inference time of all trained models on Nvidia Jetson Nano devices installed in the driving vehicle. By comparing the trade-off between detection accuracy and inference time, we have obtained a fast inference speed of 133.30 ms along with a detection performance of mAP50 0.766.

We plan to continue our future research to propose a more notable brake light status detection algorithm. One aspect of our future work involves improving the network architecture and considering sequential image inputs for more accurate and faster detection. In this study, we conducted transfer learning by keeping the YOLOv8 network structure unchanged while changing only the task for driving vehicle and brake light status detection. Therefore, we will aim to refine the network architecture to be more suitable for the task. In this study, since we utilized the input data shape of YOLOv8 without any change, it limited us to using only the current single image as the input. As mentioned in Section 3.1, it is true that preceding or succeeding images can provide valuable information for the brake light status detection. Hence, we plan to improve detection performance in future research by considering additional input images, such as preceding frames, along with the current image. Another aspect of our future work will involve conducting experimental research on the utilization of brake light status detection results for improving the safety, interpretability, and alleviation of motion sickness in autonomous driving systems. One of the objectives of proposing a fast and accurate brake light status detection in this study is to enhance the safety, interpretability, and comfort of autonomous driving technology. In conclusion, we plan to conduct empirical studies applying the brake light status detection algorithm to autonomous driving systems.

## Figures and Tables

**Table 1:** Details of the train and test datasets.

Number of		Train	Test
Images		7892	3196
Annotations		19,913	10,531
	– Brake Light Off ($C_{1}=1.0$)	10,851	5999
	– Brake Light On ($C_{2}=1.0$)	9062	4532

**Table 2:** Official deployed models of YOLOv8.

Model	Number of Params (M)	FLOPs (B)
YOLOv8n	3.2	8.7
YOLOv8s	11.2	28.6
YOLOv8m	25.9	78.9
YOLOv8l	43.7	165.2
YOLOv8x	68.2	257.8

Note: All values in the table correspond to an input image size of 640 × 640.

**Table 3:** Results of the entire test set.

Model	Class	Precision	Recall	F1 Score	mAP50	mAP50-95
YOLOv8n	Brake Light Off	0.726	0.670	0.697	0.761	0.494
	Brake Light On	0.549	0.853	0.668	0.772	0.516
	Total	0.638	0.762	0.695	0.766	0.505
YOLOv8s	Brake Light Off	0.755	0.646	0.696	0.770	0.502
	Brake Light On	0.591	0.837	0.693	0.768	0.511
	Total	0.673	0.742	0.706	0.769	0.507
YOLOv8m	Brake Light Off	0.755	0.650	0.699	0.775	0.514
	Brake Light On	0.579	0.839	0.685	0.794	0.533
	Total	0.667	0.745	0.704	0.785	0.524
YOLOv8l	Brake Light Off	0.746	0.655	0.698	0.775	0.515
	Brake Light On	0.552	0.867	0.675	0.799	0.539
	Total	0.649	0.761	0.701	0.787	0.527
YOLOv8x	Brake Light Off	0.746	0.650	0.695	0.779	0.518
	Brake Light On	0.578	0.859	0.691	0.807	0.546
	Total	0.662	0.754	0.705	0.793	0.532

**Table 4:** Details of test dataset.

Number of		Day	Night
Images		1467	1729
Annotations		4911	5620
	– Brake Light Off ($C_{1}=1.0$)	3563	2436
	– Brake Light On ($C_{2}=1.0$)	1348	3184

**Table 5:** Results of comparison for ambient illumination difference.

Model	Class	mAP50		mAP50-95	
		**Day**	**Night**	**Day**	**Night**
YOLOv8n	Brake Light Off	0.808	0.671	0.563	0.366
	Brake Light On	0.699	0.803	0.473	0.536
	Total	0.753	0.737	0.518	0.451
YOLOv8s	Brake Light Off	0.821	0.675	0.574	0.369
	Brake Light On	0.692	0.804	0.466	0.535
	Total	0.757	0.739	0.520	0.452
YOLOv8m	Brake Light Off	0.822	0.692	0.587	0.382
	Brake Light On	0.729	0.824	0.500	0.550
	Total	0.776	0.758	0.544	0.466
YOLOv8l	Brake Light Off	0.821	0.694	0.588	0.383
	Brake Light On	0.733	0.827	0.509	0.554
	Total	0.777	0.761	0.549	0.468
YOLOv8x	Brake Light Off	0.825	0.707	0.590	0.393
	Brake Light On	0.740	0.836	0.515	0.562
	Total	0.782	0.772	0.552	0.477

**Table 6:** Results of comparison for inference time in different environments. All models were inferred by converting them to ONNX form, and the different computing environments are as follows: “@GPU” refers to the Nvidia Tesla T4, “@Nano” refers to the Nvidia Jetson Nano, and “@CPU” refers to the Intel Xeon processor.

Model	Inference Time (ms) ONNX @Nano	ONNX @GPU	ONNX @CPU
YOLOv8n	12.04	133.30	353.13
YOLOv8s	12.16	217.20	891.93
YOLOv8m	20.20	471.59	1792.31
YOLOv8l	29.09	733.27	3340.26
YOLOv8x	41.87	1208.69	4222.87

**Table 7:** Comparison of learning-based brake light status detection algorithms.

Study	Proposed Work	Dataset			Brake Light Detection		Evaluation	
		**# ${}^{1}$ Data**	**# ${}^{1}$ Samples**	**Condition** ${}^{2}$	**Classification**	**Localization**	**Performance**	**Inference Time (ms)**
Cui et al. [13]	HOG, SVM	10,000 images	– ${}^{3}$	D	Turned On		0.75 (Detection rate)	N/T ${}^{4}$
Nava et al. [14]	YOLO, SVM	9700 images	9700	D	Turned On/Off		0.95 (F1 score)	N/T ${}^{4}$
Pirhonen et al. [15]	YOLOv3, RF	822 images	822	D	Turned On/Off		0.82 (Accuracy)	N/T ${}^{4}$
Wang et al. [16]	HOG, CNN	5600 images	5600	D	Turned On		0.89 (Accuracy)	N/T ${}^{4}$
Kim [18]	YOLOv4, CNN, LSTM	189 videos	– ${}^{3}$	T	Turned On/Off		0.91 (Accuracy)	N/T ${}^{4}$
Li et al. [17]	YOLOv3 tiny (w/SPP)	4618 images	15,197	D, N	Turned On	✓ ${}^{5}$	0.89 (mAP50)	15.87 (GTX-1060)
Ours	YOLOv8n	11,088 images	30,444	D, N, T	Turned On/Off	✓ ${}^{5}$	0.76 (mAP50)	12.04 (Tesla T4) 133.30 (Jetson Nano)

^1^ # = Number of. ^2^ The abbreviations for condition mean the following, respectively: D = Daytime, N = Nighttime, T = Tunnel. ^3^ – = Not reported. ^4^ N/T = Not Tested. ^5^ ✓ = Be considered.

## Data Availability

The code used in this study is available at: https://github.com/gsethan17/one-stage-brake-light-status-detection (accessed on 9 July 2023). The pre-processed training dataset is publicly available [28].

## Abbreviations

The following abbreviations are used in this manuscript:

ADAS	Advanced Driver Assistance Systems
SAE	Society for Automotive Engineers
ACC	Adaptive Cruise Control
TTC	Time to Collision
RGB	Red-Green-Blue
HSI	Hue-Saturation-Intensity
HSV	Hue-Saturation-Value
SVM	Support Vector Machine
CNN	Convolutional Neural Networks
LSTM	Long Short-Term Memory
HOG	Histogram of Oriented Gradients
SSP	Spatial Pyramid Pooling
HDR	High Dynamic Range
LED	Light Emitting Diode
CIoU	Complete Intersection over Union
DFL	Distribution Focal Loss
NMS	Nonmaximum Suppression
SGD	Stochastic Gradient Descent
NAG	Nesterov Accelerated Gradient
mAP	mean Average Precision
IoU	Intersection over Union
ONNX	Open Neural Network Exchange
